# Identification and genome characterization of genotype B and genotype C bovine parainfluenza type 3 viruses isolated in the United States

**DOI:** 10.1186/s12917-015-0431-8

**Published:** 2015-05-15

**Authors:** John D. Neill, Julia F. Ridpath, Binu T. Valayudhan

**Affiliations:** USDA, Agricultural Research Service, National Animal Disease Center, Ruminant Diseases and Immunology Research Unit, 1920 Dayton Avenue, Ames, IA 50010 USA; Texas A&M Veterinary Medical Diagnostic Laboratory, Amarillo, TX USA

**Keywords:** Deep sequencing, Virus genome, Paramyxovirus, Virus genotype, Subgenotype

## Abstract

**Background:**

Bovine parainfluenza 3 viruses (BPI3V) are respiratory pathogens of cattle that cause disease singly but are often associated with bovine respiratory disease complex (BRDC) in conjunction with other viral and bacterial agents. Bovine vaccines currently contain BPI3V to provide protection against the virus, but there is no current information regarding the BPI3V strains that are circulating in the U.S.

**Results:**

A project was initiated to sequence archival BPI3V isolates to study viral evolution over time. This was done with a deep sequencing protocol that generated sequences of multiple RNA virus genomes simultaneously. Analysis of the BPI3V sequences revealed that, in addition to the genotype A (BPI3Va) viruses previously described in the United States, there were two additional genotypes of BPI3V circulating that had been described only in Australia (BPI3Vb) and Asia (BPI3Vc). The U.S. BPI3Vb and BPI3Vc isolates showed some divergence from the Australian and Asian strains; the BPI3Vb were 93 % similar to the Australian Q5592 strain and the BPI3Vc viruses were 98 % similar to the 12Q061 strain that was described in South Korea. Overall, the three genotypes were 82 to 84 % identical to each other and 80 % identical to the most similar human PI3V. Cross-neutralization studies using an APHIS/NVSL BPI3V reference serum showed that neutralization titers against the genotype B and C viruses were 4- to ≥16-fold less then the titer against the APHIS BPI3Va reference strain, SF-4.

**Conclusions:**

This study clearly demonstrated that BPI3Vb and BPI3Vc strains, previously thought to be foreign to the U.S., are indeed circulating in domestic livestock herds. Based on virus neutralization using polyclonal antisera, there were antigenic differences between viruses from these genotypes and the BPI3Va viruses that are included in currently marketed bovine vaccines. Further study of these viruses is warranted to determine pathogenic potential and cross-protection afforded by vaccination.

## Background

Bovine parainfluenza type 3 virus (BPI3V) is a member of the *Paramyxoviridae*, genus *Respirovirus*. These viruses are respiratory pathogens of cattle. While most uncomplicated acute infections are subclinical, they can cause respiratory disease characterized by cough, fever and nasal discharge. They are among the viruses thought to contribute to bovine respiratory disease complex (BRDC; [1]).

The BPI3V genome consists of a single-stranded, negative sense RNA molecule of approximately 15,450 bases that encodes 6 large open reading frames (ORFs). The ORFs encode, from 5′ to 3′ by the positive sense strand, the nucleocapsid protein, phosphoprotein, matrix protein, fusion protein (F), hemagglutinin/neuraminidase (HN), and large polymerase protein. The F and HN proteins are glycosylated and displayed on the surface of the virus particle. Additionally, the F protein is proteolytically cleaved by the cellular protease furin to produce the mature, functional fusion protein [2]. The intergenic regions contain conserved sequences necessary for the proper initiation and termination of transcription of all open reading frames [3–5].

To date, three genotypes of BPI3V have been described. These genotypes, termed A (BPI3Va), B (BPI3Vb) and C (BPI3Vc), were differentiated based on phylogenetic analysis. Multiple BPI3Va strains have been isolated in North America [6–8] but have also been isolated in China [9], and Japan [10]. BPI3Vb was originally isolated in Australia [11] and only one full length genomic sequence has been derived. Isolations of BPI3Vc were made in China [12], South Korea [13] and Japan [14]. In addition, all three genotypes have been reported in Argentina, based on the sequences of a portion of the M protein coding sequences following PCR amplification [15].

Here, we describe the isolation and genomic sequencing of BPI3Va, BPI3Vb and BPI3Vc strains isolated from cattle in the U.S. These strains were assigned to the appropriate genotypes following phylogenetic analysis of the near complete genome sequences. The U.S. BPI3Vb and BPI3Vc strains have diverged from the Australian and South Korean viruses, indicating they were present in the U.S. for some time before the isolation of these viruses.

## Results

### Sequencing and assembly of BPI3V genomes

The sequences derived from the U.S. isolates of BPI3V were assembled using full-length BPI3V genomic sequences from GenBank as template. Because viruses of genotype A were the only BPI3V reported in the U.S., a representative genotype A virus was used initially. This resulted in extremely poor assemblies. One contiguous sequence that did assemble was used in a BLAST search of GenBank that resulted in the identification of the closest match to be a BPI3Vb (Q5592). All sequence data sets were then assembled using templates from each of the 3 BPI3V genotypes. From these assemblies, two BPI3Vb (TMVDL15 and TMVDL17), two BPI3Vc (TMVDL16 and TMVDL20) and two BPI3Va (TMVDL24 and TMVDL60) were identified.

The number of individual sequences from each library ranged from 33,393 to 85,159 (Table 1). The ratio of virus to total sequences was highly dependent on the titer of the virus in the original samples [16]. The percentage of viral sequences in each library ranged from 7.3 to 59.6. The average depth of coverage for the 6 viruses ranged from 54.0 to 386.9x. Because this sequencing protocol did not result in assembly of full-length genomic sequences, PCR was used to derive these sequences to yield full genome sequences. All genome lengths conformed to the paramyxovirus rule of six [17, 18].

**Table 1:** Number of sequences obtained for six BPI3V isolates

Virus	TVMD15	TVMD16	TVMD17	TVMD20	TVMD24	TVMD60
Total sequences	85,159	33,393	83,045	33,832	77,033	62,393
Virus sequences	6175 (7.3)^a^	19,897 (59.6)	48,609 (58.5)	5556 (16.4)	15,343 (19.9)	29,686 (47.6)
Ave. Depth (%)	59.7	162.6	386.9	54.0	134.6	280.7

^a^percent viral sequences

### BPI3V genomic relationships

**Figure 1:**
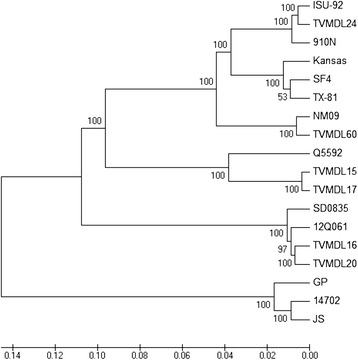
Phylogenetic tree of BPI3V sequences and comparison to human PI3V sequences from GenBank

The genomic RNA sequences of the six viruses from this study, as well as full-length genomic sequences of BPI3V obtained from GenBank were aligned and a phylogenetic tree derived (Table 2). The resulting dendrogram showed that these viruses formed three very distinct subgroups (Fig. 1). Three human PI3V (HPI3V) sequences were included for comparison. Interestingly, BPI3Va were the most genetically diverse group, forming what appeared to be three distinct subgroups. The three BPI3Va viruses were 91.4 to 94.3 % identical between groups and 96.9 to 99 % identical within groups. TMVDL24 and TMVDL60 were most closely related to ISU92 and NM09, respectively. The APHIS reference strain SF-4 was found in the third BPI3Va subgroup. Similarly, as shown in Fig. 1, the BPI3Vb appeared to form two subgroups, where the U.S. isolates constituted one subgroup and the Australian isolate Q5592 another. The two U.S. isolates were 99.3 % identical to each other while being only 92.7 % identical to Q5592. The BPI3Vc comprised the most homogeneous genotype. The two Asian isolates were 97.7 % identical, and the two U.S. isolates were 98.7 % identical to each other, with slightly greater identity with the South Korean 12Q061 isolate. Table 3 shows the pairwise comparison of 15 strains of BPI3V. The percent identities range from 80.9 % (12Q061 and Q5592) to 99 % identical (ISU92 and TMVDL24).

**Table 2:** Viruses uses in this study

Virus	GenBank accession #	Year of isolation	Genotype	Country	Genome length
ISU92	EU439428	1992	A	USA	15480
Kansas	AF178654	1984	A	USA	15456
NM09	JQ063064	2009	A	China	15456
910 N	D84095	Unknown	A	Japan	15480
SF4	AF178655	1958	A	USA	15456
TX81	EU439429	1981	A	USA	15456
TVMD24	KJ647288	2008	A	USA	15480
TVMD60	KJ647289	2007	A	USA	15456
Q5592	EU277658	Unknown	B	Australia	15498
TVMD15	KJ647284	2009	B	USA	15474
TVMD17	KJ647286	2007	B	USA	15474
12Q061	JX969DD1	2012	C	S. Korea	15474
SD0835	HQ530153	2008	C	China	15474
TVMD16	KJ647285	2007	C	USA	15474
TVMD20	KJ647287	2012	C	USA	15474
GP	ABD12132	Unknown	human	Japan	15462
JS	Z11575	Unknown	human	USA	15462
14702	EU426062	Unknown	human	Canada	15462

**Table 3:** Pairwise comparison of BPI3V full-length genomic sequences

	Percent identity														
	SF4	Kansas	TX81	910 N	ISU92	TVMD24	NM09	TVMD60	Q5592	TVMD15	TVMD17	SD0835	12Q061	TVMD16	TVMD20
SF4	*	98.2	98.2	92.5	92.7	92.4	92.2	92.1	83.5	83.8	83.8	82.3	82.1	82.4	82.3
Kansas		*	96.9	92.4	92.5	92.3	92.2	92	83.6	83.9	83.9	82.4	82.2	82.5	82.4
TX81			*	93.8	94.3	93.9	92	91.9	83.3	83.7	83.8	82.5	82.2	82.5	82.4
910 N				*	98.1	98.5	91.6	91.6	83	83.6	83.6	82.1	81.7	81.9	81.9
ISU92					*	99	91.4	91.4	82.9	83.4	83.5	82	81.7	81.9	81.9
TVMD24						*	91.6	91.6	82.9	83.5	83.5	82.1	81.6	81.9	81.9
NM09							*	98.8	82.8	83.3	83.3	82.1	81.9	82.2	82.1
TVMD60								*	82.9	83.5	83.4	82.1	81.9	82.3	82.1
Q5592									*	92.8	92.7	81.1	80.9	81.2	81.1
TVMD15										*	99.3	81.3	81.2	81.4	81.4
TVMD17											*	81.4	81.2	81.4	81.4
SD0835												*	97.7	98.1	97.9
12Q061													*	98.4	98.1
TVMD16														*	98.7
TVMD20															*

### Neutralization of BPI3V isolates

Serum neutralization assays were conducted using the APHIS BPI3Va reference antiserum to neutralize the APHIS BPI3V reference SF-4 and the six field isolates of BPI3V. The neutralization titer of the serum against SF-4 was 1:128 (Table 4). The same antiserum had neutralization titers that ranged from < 8 (TVMDL15 and 24) to 1:32 (TVMDL20) against U.S. strains. The reference serum reacted more strongly against the BPI3Vc then against the BPI3Vb and to the other BPI3Va, but still had a titer against SF-4 that was 4-fold higher than against TVMDL20. These results suggest that significant antigenic differences may exist between the different genotypes.

**Table 4:** Virus neutralization assays of BPI3V strains using APHIS 475 BDV 0601 reference serum

	Virus strain						
	SF-4	TVMDL15	TVMDL16	TVMDL17	TVMDL20	TVMDL24	TVMDL60
Antibody titer^a^	128	<8	16	8	32	<8	8

^a^reciprocal of highest dilution giving complete neutralization

## Discussion

BPI3V strains described here were part of a collection of BPI3V isolates ranging from 1985 to the present. Until now, only the genotype A BPI3V was known to be present in the U.S. Sequencing of these recent isolates revealed that genotypes previously described in Australia and Asia were also present. This was unanticipated because of the total absence of evidence for additional genotypes. Additionally, this study demonstrated that BPI3Va strains are still in circulation. Diagnostic reagents used in this country are based on sequences and antisera generated with BPI3Va, which may reduce detection of other genotypes.

A recent report showed that these three BPI3V genotypes were present in water buffalo populations in Argentina [15] with this being the only description of BPI3Vb and BPI3Vc outside of Australia and Asia. The sequence data reported here do not provide information regarding timing of introduction of these genotypes into this country. There was more divergence of the BPI3Vb from the Australian isolate than BPI3Vc from the Asian isolates, indicating a possible earlier introduction. Overall, the BPI3Vc was the most homogeneous, indicating a closer timeline.

Phylogenetic comparison of the genomic sequences of viruses from the three BPI3V genotypes provided evidence that two of the genotypes could be further subdivided into subgenotypes based on degree of divergence. The BPI3Va could be divided into three subgroups. The two BPI3Va strains reported here belonged to two distinct subgroups while the SF-4 reference strain was assigned to the third. These similarities were reported previously [19], where two distinct subgenotypes were formed by ISU92 and 910 N and by SF4 and TX-81. Here, we demonstrated that there is a third subgenotype formed by NM09 and TVLMD60. Similarly, PBI3Vb appears to form two subgenotypes, however, this needs to be confirmed by further sequencing of the archival viruses. If this is shown to be true, it can perhaps be explained by the geographic separation of the viruses over an unknown length of time. However, it is more difficult to explain the presence of 3 subgenotypes of BPI3Va where all were all in circulation within the U.S. without discernable temporal or geographic separation.

An earlier study described the isolation and characterization of two porcine isolates of BPI3Va, TX81 and ISU92 [7, 19]. It was proposed that these viruses were variants of BPI3Va that were transferred to pigs from cattle. The authors demonstrated that these viruses had low pathogenicity in pigs as well as low sero-prevalence in domestic swine herds. Based on the amino acid sequence, TX81 was believed to be a true transfer from cattle but ISU92 was considered a subspecies and adapting to swine. Data presented here clearly shows that viruses closely related to ISU92 are circulating in cattle herds, suggesting that ISU92 resulted from a transfer of the bovine virus rather than a new virus arising via adaptation to growth in swine. Additionally, sequencing of other BPI3V isolates in the archival collection revealed that TX81-like strains are also found in circulation in domestic cattle herds (data not shown). There is no evidence for species shift in these viruses, because of the isolation of closely related viruses from cattle.

There is currently no data concerning differences in pathogenicity of the different genotypes in cattle. It is unknown how the genotypes and the potential subgenotypes differ in pathogenic potential or antigenic characteristics, but the preliminary serological data presented here indicates that there may be differences in antigenicity. This observation is supported by data presented by a previous neutralization study [15] that showed there was differences in neutralization of SF4 and a water buffalo BPI3Vb isolate using water buffalo antisera. This raises questions about the efficacy of BPI3V vaccines presently used in this country. All vaccines currently in use contain BPI3Va strains. Data from sequencing additional isolates from the archival collection of BPI3V indicates that BPI3Va are now only a minority and that members of all three genotypes and their subgenotypes are in circulation (data not shown). Knowledge of the variants and genotypes of BPI3V in domestic livestock herds is necessary for the design of the most efficacious vaccines, such as has been demonstrated for bovine viral diarrhea virus [20]. This information is also necessary to develop rapid and accurate diagnostic tests capable of detecting all BPI3V strains. We have demonstrated the presence of BPI3Vb and BPI3Vc viruses in the U.S., the next step will be to develop diagnostic reagents to detect all BPI3V strains in circulation in domestic livestock herds.

## Methods

### Viruses, RNA extraction and sequencing

Six viral strains isolated from clinical samples from respiratory disease outbreaks submitted to the Texas A&M Veterinary Diagnostic Laboratory between 2007 and 2012 were used in the study. These viruses were isolated by serial passage in bovine turbinate cells (ATCC CRL-1390) using standard procedures of virus isolation in cell culture. The viruses were confirmed as BPI3V by fluorescent antibody staining [21]. After growth of the virus on the appropriate cell type, the medium was frozen/thawed thrice, cell debris removed by centrifugation, and aliquots frozen at −80 °C until further use. The virus stocks were treated with a nuclease cocktail, viral RNA isolated and sequencing libraries composed of 20 viral genomes were prepared and sequenced using the Ion Torrent PGM platform as previously described [16].

### Sequence analysis and virus genome assembly

The Ion Torrent raw data files containing the sequence data from each sequencing run of the PGM were demultiplexed using the 20 base sequence of the random primer. The resulting sequence files were then used to assemble near full length genomic sequences using SeqManNGen and were edited with the SeqMan software, both of the Lasergene 10 package (DNAstar, Inc., Madison, WI). Related viral sequences obtained from GenBank were used as assembly reference sequences. Assembled genomic sequences were compared using Aligner (Codoncode, Inc., Centerville, MA). Phylogenetic trees were done using MEGA ver. 5.1 [22]. The GenBank accession numbers and country of origin of all viruses used in this study are shown in Table 2.

### Genomic termini and intergenic region sequencing

The genome termini (25 to 200 terminal bases) were generally not found in the Ion Torrent sequencing data, likely due to the probable presence of stable secondary structures. PCR was used to amplify the termini from each virus to obtain these sequences (Table 5). The PCR and sequencing was done as previously described [23].

**Table 5:** PCR primers used in this study

BPI3V 3′ minus	ACCAAACAAGAGAAAAACTCTGTTTGG
Genotype A 3′ plus	AATTGGAGATGATGACGTTGTTCTAGTATC
Genotype B 3′ plus	GATCGGAGACGATGACGTAGTTCTGGTATC
Genotype C 3′ plus	AGTTGGAGATGATGATGTTATCTTGGTATC
BPI3V 5′ plus	ACCAAACAAGAGAAGAGACTTG
Genotype A & B 5′ minus	ACATATTTAACATCTGCATTACTACC
Genotype C 5′ minus	ACATATTTGACATCTGCATTATTACC
Genotype B F/HN plus	TGGGAGCTGGTACCAGTCAAGTGC
Genotype B F/HN minus	ACCAATTCCTTCAGAGGTCTCTTG
Genotype C F/HN plus	CTCTGTTGGAGGTTGGTATCAGTC
Genotype C F/HN minus	CCAATTTCATCTGCAGCTTGCTGG
Genotype B HN/L plus	TAACAACAGGACACTTCCAGCCGC
Genotype B HN/L minus	TTTACCCTTTCCATCAGGACAGAC
Genotype C HN/L plus	CATCACTTACGCAACAGACACACG
Genotype C HN/L minus	TTAATTCCAGGTATACACAATTGG

Sequences within some M/F and F/HN intergenic regions were found to contain homopolymeric stretches of adenines. Due to the propensity of the Ion Torrent data for Indels associated with homopolymeric stretches of nucleotides [24, 25], these regions were difficult to assemble accurately in the genotype B and C viruses. Genotype-specific PCR primers were designed to amplify these intergenic regions for Sanger sequence analysis (Table 5). PCR and sequencing was conducted as described above.

### Virus neutralization assays

Virus neutralization assays (VNs) were done as previously described [26]. An Animal and Plant Health Inspection Service (APHIS) BPI3V reference antiserum raised against SF-4 (#475 BDV 0601) and APHIS reference virus BPI3V, SF-4, were used to compare neutralization titers against BPI3Vb and BPI3Vc strains.

## Conclusions

Sequence analysis of the genomic RNAs of six BPI3V strains isolated in the U.S. between 2007 and 2012 revealed that two genotypes of the virus thought to be exotic to this country are indeed present and circulating in domestic livestock herds. BPI3Vb and BPI3Vc strains show some divergence from that reported for viruses isolated in Australia and Asia, respectively. VNs using an APHIS reference antiserum raised against SF-4, a BPI3Va strain, suggested that significant differences exist in the antigenic characteristics of these viruses. Evaluation of the protection afforded against the genotype B and C viruses by vaccines currently marketed in the U.S. is warranted.

## Abbreviations

BPI3V: Bovine parainfluenza virus type 3
BPI3Va,b,c: Bovine parainfluenza virus type 3 genotype A, B or C
BRDC: Bovine respiratory disease complex
F: Fusion protein
HN: Hemaglutinin/neuraminidase protein
Indel: Insertion or deletion
VN: Virus neutralization

## References

[1] Ellis JA (2010). Bovine parainfluenza-3 virus. Vet Clin North Am Food Anim Pract.

[2] Ortmann D, Ohuchi M, Angliker H, Shaw E, Garten W, Klenk HD (1994). Proteolytic cleavage of wild type and mutants of the F protein of human parainfluenza virus type 3 by two subtilisin-like endoproteases, furin and Kex2. J Virol.

[3] Bousse T, Matrosovich T, Portner A, Kato A, Nagai Y, Takimoto T (2002). The long noncoding region of the human parainfluenza virus type 1 f gene contributes to the read-through transcription at the m-f gene junction. J Virol.

[4] Bousse T, Takimoto T, Murti KG, Portner A (1997). Elevated expression of the human parainfluenza virus type 1 F gene downregulates HN expression. Virology.

[5] Power UF, Ryan KW, Portner A (1992). Sequence characterization and expression of the matrix protein gene of human parainfluenza virus type 1. Virology.

[6] Reisinger RC, Heddleston KL, Manthei CA (1959). A myxovirus (SF-4) associated with shipping fever of cattle. J Am Vet Med Assoc.

[7] Qiao D, Janke BH, Elankumaran S (2009). Molecular characterization of glycoprotein genes and phylogenetic analysis of two swine paramyxoviruses isolated from United States. Virus Genes.

[8] Bailly JE, McAuliffe JM, Skiadopoulos MH, Collins PL, Murphy BR (2000). Sequence determination and molecular analysis of two strains of bovine parainfluenza virus type 3 that are attenuated for primates. Virus Genes.

[9] Wen YJ, Shi XC, Wang FX, Wang W, Zhang SQ, Li G (2012). Phylogenetic analysis of the bovine parainfluenza virus type 3 from cattle herds revealing the existence of a genotype A strain in China. Virus Genes.

[10] Ohkura T, Kokuho T, Konishi M, Kameyama K, Takeuchi K. Complete Genome Sequences of Bovine Parainfluenza Virus Type 3 Strain BN-1 and Vaccine Strain BN-CE. Genome Announc 2013, 1(1)): doi:10.1128/genomeA.00247-12. 10.1128/genomeA.00247-12 PMC3587952 23469358

[11] Horwood PF, Gravel JL, Mahony TJ (2008). Identification of two distinct bovine parainfluenza virus type 3 genotypes. J Gen Virol.

[12] Zhu YM, Shi HF, Gao YR, Xin JQ, Liu NH, Xiang WH (2011). Isolation and genetic characterization of bovine parainfluenza virus type 3 from cattle in China. Vet Microbiol.

[13] Oem JK, Lee EY, Lee KK, Kim SH, Lee MH, Hyun BH (2013). Molecular characterization of a Korean bovine parainfluenza virus type 3 isolate. Vet Microbiol.

[14] Konishi M, Ohkura T, Shimizu M, Akiyama M, Kameyama K, Takeuchi K. Complete genome sequence of the first isolate of genotype C bovine parainfluenza virus type 3 in Japan. Genome announcements 2014, 2(6): doi: 10.1128/genomeA.01215-14. 10.1128/genomeA.01215-14 PMC4246162 25428970

[15] Maidana SS, Lomonaco PM, Combessies G, Craig MI, Diodati J, Rodriguez D (2012). Isolation and characterization of bovine parainfluenza virus type 3 from water buffaloes (Bubalus bubalis) in Argentina. BMC Vet Res.

[16] Neill JD, Bayles DO, Ridpath JF (2014). Simultaneous Rapid Sequencing of Multiple RNA Virus Genomes. J Virol Methods.

[17] Kolakofsky D, Pelet T, Garcin D, Hausmann S, Curran J, Roux L (1998). Paramyxovirus RNA synthesis and the requirement for hexamer genome length: the rule of six revisited. J Virol.

[18] Calain P, Roux L (1993). The rule of six, a basic feature for efficient replication of Sendai virus defective interfering RNA. J Virol.

[19] Qiao D, Janke BH, Elankumaran S (2010). Complete genome sequence and pathogenicity of two swine parainfluenzavirus 3 isolates from pigs in the United States. J Virol.

[20] Ridpath JF, Lovell G, Neill JD, Hairgrove TB, Velayudhan B, Mock R (2011). Change in predominance of Bovine viral diarrhea virus subgenotypes among samples submitted to a diagnostic laboratory over a 20-year time span. J Vet Diagn Invest.

[21] Leland D, Landry ML, Jerome KR (2010). Virus Isolation. Lennette’s Laboratory Diagnosis of Viral Infections.

[22] Tamura K, Peterson D, Peterson N, Stecher G, Nei M, Kumar S (2011). MEGA5: molecular evolutionary genetics analysis using maximum likelihood, evolutionary distance, and maximum parsimony methods. Mol Biol Evol.

[23] Neill JD, Newcomer BW, Marley SD, Ridpath JF, Givens MD (2011). Genetic change in the open reading frame of bovine viral diarrhea virus is introduced more rapidly during the establishment of a single persistent infection than from multiple acute infections. Virus Res.

[24] Bragg LM, Stone G, Butler MK, Hugenholtz P, Tyson GW (2013). Shining a light on dark sequencing: characterising errors in Ion Torrent PGM data. PLoS Comput Biol.

[25] Yeo ZX, Chan M, Yap YS, Ang P, Rozen S, Lee AS (2012). Improving indel detection specificity of the Ion Torrent PGM benchtop sequencer. PLoS One.

[26] anon (1978). Paramyxoviruses. Manual of Standardized Methods for Veterinary Microbiology.

